# Dual Blockade of TGF-β Receptor and Endothelin Receptor Synergistically Inhibits Angiotensin II-Induced Myofibroblast Differentiation: Role of AT_1_R/G_αq_-Mediated TGF-β1 and ET-1 Signaling

**DOI:** 10.3390/ijms24086972

**Published:** 2023-04-09

**Authors:** Ratchanee Duangrat, Warisara Parichatikanond, Supachoke Mangmool

**Affiliations:** 1 Department of Pharmacology, Faculty of Science, Mahidol University, Bangkok 10400, Thailand; 2 Molecular Medicine Graduate Program, Faculty of Science, Mahidol University, Bangkok 10400, Thailand; 3 Department of Pharmacology, Faculty of Pharmacy, Mahidol University, Bangkok 10400, Thailand; 4 Centre of Biopharmaceutical Science for Healthy Ageing (BSHA), Faculty of Pharmacy, Mahidol University, Bangkok 10400, Thailand

**Keywords:** α-SMA, angiotensin II, angiotensin receptor blocker (ARB), cardiac fibrosis, endothelin-1, endothelin receptor antagonist (ERA), myofibroblast differentiation, TGF-β1

## Abstract

Angiotensin II (Ang II) upregulates transforming growth factor-beta1 (TGF-β1) and endothelin-1 (ET-1) in various types of cells, and all of them act as profibrotic mediators. However, the signal transduction of angiotensin II receptor (ATR) for upregulation of TGF-β1 and ET-1, and their effectors that play an essential role in myofibroblast differentiation, are not fully understood. Therefore, we investigated the ATR networking with TGF-β1 and ET-1 and identified the signal transduction of these mediators by measuring the mRNA expression of alpha-smooth muscle actin (α-SMA) and collagen I using qRT-PCR. Myofibroblast phenotypes were monitored by α-SMA and stress fiber formation with fluorescence microscopy. Our findings suggested that Ang II induced collagen I and α-SMA synthesis and stress fiber formation through the AT_1_R/G_αq_ axis in adult human cardiac fibroblasts (HCFs). Following AT_1_R stimulation, G_αq_ protein, not G_βγ_ subunit, was required for upregulation of TGF-β1 and ET-1. Moreover, dual inhibition of TGF-β and ET-1 signaling completely inhibited Ang II-induced myofibroblast differentiation. The AT_1_R/G_αq_ cascade transduced signals to TGF-β1, which in turn upregulated ET-1 via the Smad- and ERK1/2-dependent pathways. ET-1 consecutively bound to and activated endothelin receptor type A (ET_A_R), leading to increases in collagen I and α-SMA synthesis and stress fiber formation. Remarkably, dual blockade of TGF-β receptor and ETR exhibited the restorative effects to reverse the myofibroblast phenotype induced by Ang II. Collectively, TGF-β1 and ET-1 are major effectors of AT_1_R/G_αq_ cascade, and therefore, negative regulation of TGF-β and ET-1 signaling represents a targeted therapeutic strategy for the prevention and restoration of cardiac fibrosis.

## 1. Introduction

Cardiac fibrosis is a strong indicator of adverse clinical outcome in heart diseases, including heart failure (HF). In patients with HF, the degree of cardiac fibrosis is correlated with cardiac adverse events and associated with high rates of mortality and morbidity [1,2]. Angiotensin II (Ang II), a major component of the renin-angiotensin system (RAS), is involved in the pathogenesis of cardiac fibrosis by overstimulating angiotensin II receptors (ATRs). Following Ang II stimulation, cardiac fibroblasts undergo transdifferentiation into active myofibroblasts by a process called myofibroblast differentiation [3,4]. Myofibroblast is characterized by overexpression of alpha-smooth muscle actin (α-SMA) and stress fiber formation [5].

Numerous studies have shown that Ang II induced detrimental effects and pathologic changes in cardiac functions mainly through the angiotensin II type 1 receptors (AT_1_Rs) [4,6]. In contrast, stimulation of angiotensin II type 2 receptors (AT_2_Rs) exhibited cardioprotective effects, including vasodilating effects and tissue repair [7]. Although the majority of Ang II effects are mediated through AT_1_R stimulation, the AT_2_R subtype is also important due to the findings that both subtypes are upregulated in heart diseases [8,9]. ATRs are members of the G protein-coupled receptor (GPCR) superfamily, which couple with the heterotrimeric G_q_ protein. Binding of Ang II to ATRs leads to activation and dissociation of the G_αq_ protein from the G_βγ_ subunit. The activated G_αq_ protein and G_βγ_ subunit consequently interact and activate their targeted effectors [10]. However, which subtype of ATRs and subunit of G proteins mediate Ang II-induced myofibroblast differentiation in adult human cardiac fibroblasts (HCFs) have not been comprehensively identified.

The signal transduction of Ang II is complex and involves a series of molecular interactions that transmit the signal from upstream to downstream effectors. Transforming growth factor-beta1 (TGF-β1), a potent profibrotic mediator, has been implicated in ATR signaling. Stimulation of AT_1_Rs induced TGF-β1 synthesis and secretion in cardiac fibroblasts, and upregulation of TGF-β1 is necessary for Ang II-mediated hypertrophy and fibrosis [11,12]. The canonical Smads and non-canonical mitogen-activated protein kinase (MAPK) proteins are the major downstream effectors of the Ang II/TGF-β1 signaling cascade in the cardiovascular system [12,13]. Furthermore, treatment with TGF-β1 induced endothelin-1 (ET-1) synthesis in vascular endothelial cells [14,15] through the Smad signaling pathway [16]. Administration of losartan, an angiotensin receptor blocker (ARB), resulted in the inhibition of Smad2 and extracellular signal-regulated kinase (ERK) phosphorylation in Loeys–Dietz syndrome mice, contributing to aortic aneurysms [17]. Even though TGF-β1 transduces the signals through Smad- and MAPK-dependent pathways, the precise downstream effectors of TGF-β1 underlying the induction of myofibroblast differentiation needed to be investigated.

A strong correlation between Ang II and endothelin-1 (ET-1) has been demonstrated in previous studies. For instance, Ang II induced ET-1 gene expression [18]. Blockade of endothelin receptor type A (ET_A_R) abolished Ang II-increased ET-1 contents in aorta and femoral artery [19]. Treatment with losartan inhibited Ang II-induced tissue ET-1 synthesis, suggesting that blockade of AT_1_R prevents Ang II-induced upregulation of ET-1 [20]. ETRs are classified as GPCRs that have two subtypes, ET_A_R and ET_B_R [21]. ET-1 induced myofibroblast differentiation and collagen matrix contraction through the ET_A_Rs, but not ET_B_Rs, in lung fibroblasts [22]. Stimulation of ET_A_Rs led to the upregulation of collagen deposition in the hearts of hypertensive rats [23]. In addition, both subtypes of ETRs were required for ET-1-induced cell proliferation of lung fibroblasts [24]. However, it remains to be elucidated which ETR subtype is involved in ET-1-induced myofibroblast differentiation of adult HCFs. 

Inhibition of TGF-β signaling using anti-TGF-β1 antibody was found to alleviate myocardial fibrosis and cardiac abnormality in pressure-overloaded rat hearts [25]. Moreover, blockade of TGF-β receptor type I (TβRI) diminished TGF-β1-induced collagen synthesis in cardiac fibroblasts and attenuated the progression of myocardial fibrosis in a mouse model of pressure overload [26]. Bosentan, a nonselective ETR antagonist (ERA), has been shown to improve cardiac function, reduce infarct size, and attenuate myocardial fibrosis and remodeling in rats with ischemia/reperfusion injury [27]. Therefore, negative regulation of Ang II, TGF-β1 and ET-1 transmitting signals has been thought to be a potential strategy for inhibition of cardiac fibrosis. Nonetheless, the effects of TβRI/II inhibitor and ERA on the inhibition of Ang II-induced myofibroblast differentiation in adult HCFs are unknown.

So far, many HF patients have been reported to receive clinical attention only after significant fibrosis has already occurred. Therefore, the reversal of the myofibroblast phenotype may serve as a means to delay the pathological features of cardiac fibrosis. In the present study, we also investigated the restorative effects of valsartan, LY2109761 (TβRI/II inhibitor), and bosentan on myofibroblast differentiation induced by Ang II. The essential roles of TGF-β1, ET-1, and Ang II networking and identification of signal transduction of these profibrotic mediators will assist us in discovering therapeutic strategies for the treatment and prevention of cardiac fibrosis. 

## 2. Results

### 2.1. Treatment with Ang II, TGF-β1, or ET-1 Promotes Myofibroblast Differentiation in a Dose-Dependent Manner in Adult Human Cardiac Fibroblasts (HCFs)

In this study, we first determined the effects of Ang II, TGF-β1, and ET-1 on myofibroblast differentiation by measuring collagen I and α-SMA expression and stress fiber formation. Treatment with Ang II, TGF-β1, or ET-1 induced the synthesis of collagen I and α-SMA in a dose-dependent manner with maximal effects observed at 200 nM, 1 ng/mL, and 20 nM, respectively (Supplementary Figure S1A,B). Furthermore, treatment of adult HCFs with these profibrotic mediators resulted in a significant increase in stress fiber formation, as shown in the red color, in a dose-dependent fashion (Supplementary Figure S1C). Our results suggested that Ang II, TGF-β1, and ET-1 have the ability to induce the transdifferentiation of fibroblasts into myofibroblasts in adult HCFs. 

### 2.2. Ang II Induces Myofibroblast Differentiation through AT_1_R/G_αq_ Axis

**Figure 1:**
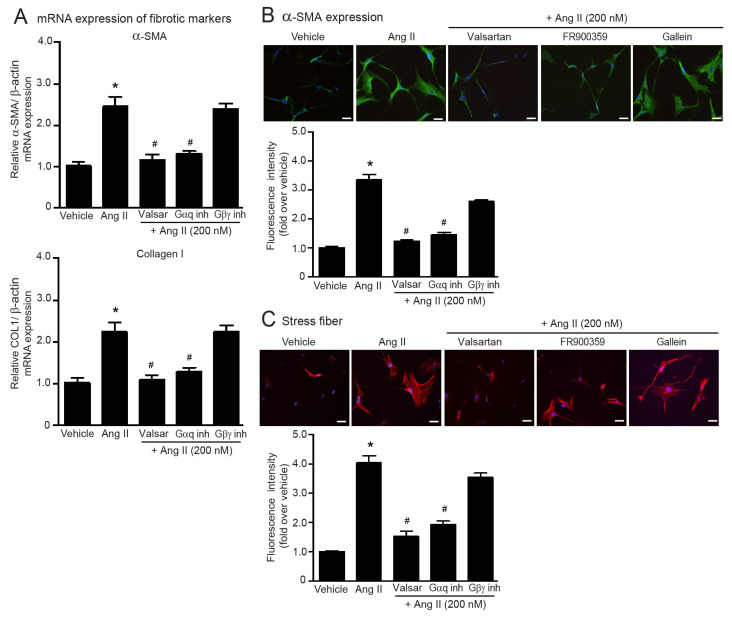
Blockade of AT_1_R and G_αq_ protein antagonizes Ang II-induced myofibroblast differentiation. Serum-starved adult HCFs were pretreated with valsartan (valsar; 1 µM), FR900359 (G_αq_ inh; 1 µM), or gallein (G_βγ_ inh; 10 µM) for 1 h before stimulation with 200 nM Ang II for 6 h (**A**) or 24 h (**B**,**C**). (**A**) Relative mRNA levels of fibrotic markers, α-SMA and collagen I, were analyzed by qRT-PCR. Data are expressed as the mean ± SEM (*n* = 4). After treatment, immunofluorescence staining was used to determine α-SMA expression (green) (**B**) and stress fiber formation (red) (**C**). Nuclei were stained with DAPI (blue). Scale bar, 10 μm. Data are expressed as the mean ± SEM (*n* = 3). * *p* < 0.05 vs. vehicle; ^#^ *p* < 0.05 vs. Ang II.

Next, we aimed to identify the signal transduction of ATRs responsible for Ang II-induced myofibroblast differentiation in adult HCFs. Pretreatment with valsartan (AT_1_R antagonist) completely blocked Ang II-induced overexpression of collagen I and α-SMA as well as stress fiber formation (Figure 1A–C). AT_1_R is a G_q_ protein-coupled receptor (G_q_PCR) that transduces its signal through both G_αq_ and G_βγ_ subunits [10]. We applied FR900359 (G_αq_ inhibitor) and gallein (G_βγ_ inhibitor) to investigate which subunits of the G protein play an essential role in AT_1_R-mediated fibrogenic effects and found that pretreatment with FR900359 inhibited Ang II-induced myofibroblast differentiation, while blockade of G_βγ_ activity using gallein did not interfere with Ang II actions (Figure 1A–C). Together, these findings indicated that Ang II exerts fibrogenic effects in AT_1_R-dependent activation of G_αq_ protein. 

### 2.3. Dual Blockade of TGF-β Receptor and Endothelin Receptor Inhibits Ang II-Induced Myofibroblast Differentiation

**Figure 2:**
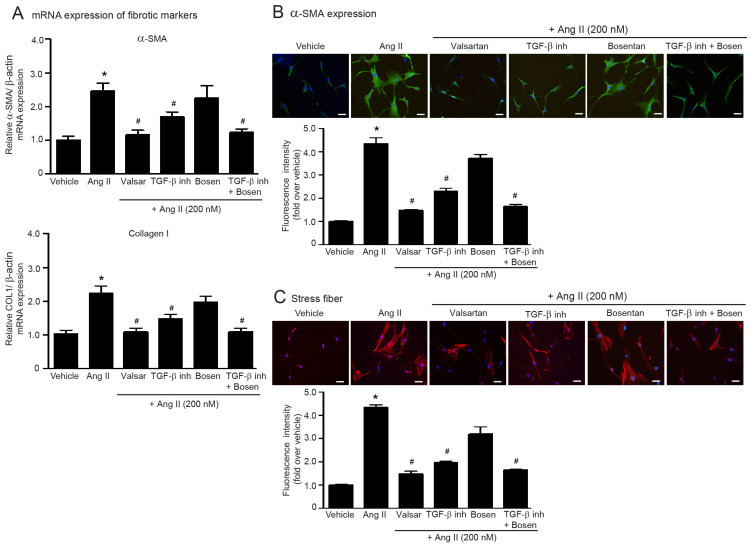
The synergistic function of TGF-β and ET-1 is required for Ang II-induced myofibroblast differentiation. Serum-starved adult HCFs were pretreated with valsartan (valsar; 1 µM), LY2109761 (TGF-β inh; 5 µM), bosentan (1 µM), or LY2109761 plus bosentan for 1 h before stimulation with 200 nM Ang II for 6 h (**A**) or 24 h (**B**,**C**). (**A**) Relative mRNA levels of fibrotic markers, α-SMA and collagen I, were analyzed by qRT-PCR. Data are expressed as the mean ± SEM (*n* = 4). After treatment, immunofluorescence staining was used to determine α-SMA expression (green) (**B**) and stress fiber formation (red) (**C**). Nuclei were stained with DAPI (blue). Scale bar, 10 μm. Data are expressed as the mean ± SEM (*n* = 3). * *p* < 0.05 vs. vehicle; ^#^ *p* < 0.05 vs. Ang II.

TGF-β1 and ET-1 are downstream effectors of Ang II and have been associated with ATR signaling. In rat aorta, Ang II induced the upregulation of TGF-β1 [11] and ET-1 protein expression [20]. Next, we used LY2109761 (TβRI/II inhibitor) and bosentan (ETR antagonist; ERA) to investigate the contributions of TGF-β1 and ET-1 to Ang II-induced myofibroblast differentiation. As shown in Figure 2, blockade of TGF-β signaling with LY2109761 suppressed Ang II-induced collagen I and α-SMA synthesis and stress fiber formation. However, LY2109761 had less inhibitory effects than valsartan. In addition, blockade of ETR signaling using bosentan did not show significant preventive effects on Ang II-induced myofibroblast differentiation. Interestingly, cotreatment of LY2109761 with bosentan completely reduced fibrogenic effects of Ang II, which had an inhibitory effect similar to those of valsartan (Figure 2A–C). These results suggested that ET-1 appears to function synergistically with TGF-β1 to promote myofibroblast differentiation induced by Ang II. Thus, dual blockade of TGF-β and ET-1 is required to prevent Ang II-induced myofibroblast differentiation in adult HCFs.

### 2.4. Blockade of ETRs, Not ATRs, Inhibits TGF-β1-Induced Myofibroblast Differentiation

**Figure 3:**
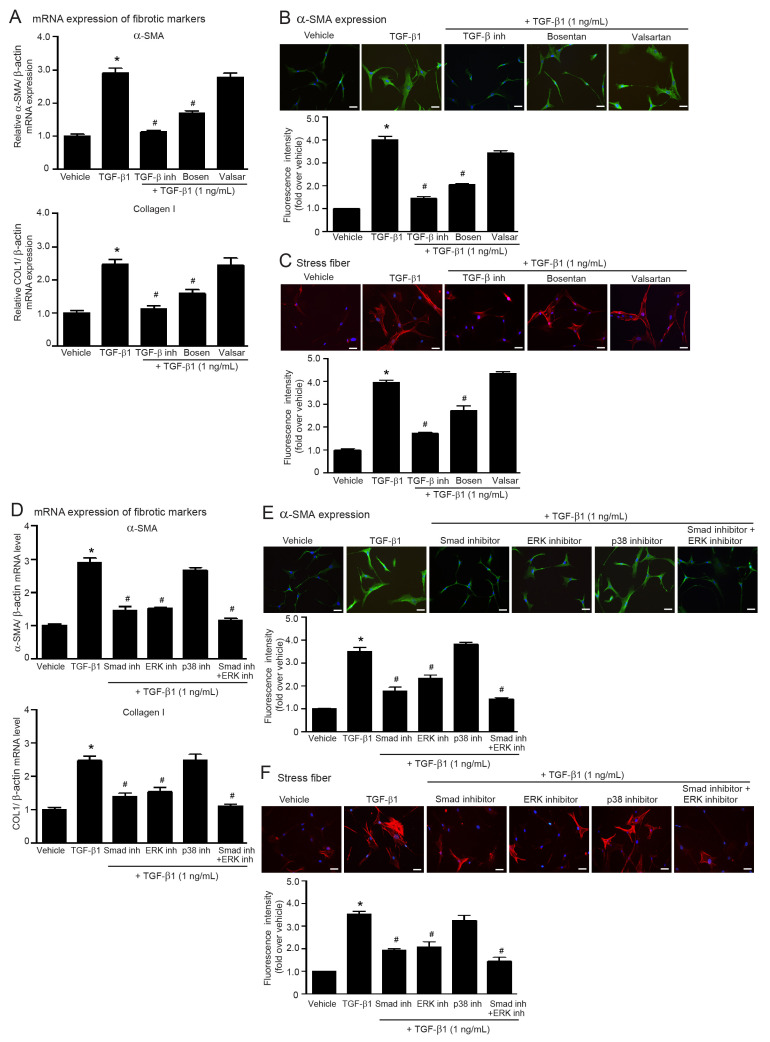
ET-1 is a downstream effector of TGF-β1, and blockade of either Smad or ERK1/2 activities attenuates profibrotic effects of TGF-β1. (**A**–**C**) Serum-starved adult HCFs were pretreated with LY2109761 (TGF-β inh; 5 µM), bosentan (1 µM), or valsartan (valsar; 1 µM) for 1 h before treatment with 1 ng/mL TGF-β1 for 6 h (**A**) or 24 h (**B**,**C**). (**D**–**F**) Serum-starved adult HCFs were pretreated with 1 µM Smad inhibitor (Smad inh), 1 µM FR180204 (ERK inhibitor; ERK inh), 1 µM SB203580 (p38 MAPK inhibitor; p38 inh), or Smad inh plus ERK inh for 1 h before treatment with 1 ng/mL TGF-β1 for 6 h (**D**) or 24 h (**E**,**F**). (**A**,**D**) Relative mRNA levels of fibrotic markers, α-SMA and collagen I, were analyzed by qRT-PCR. Data are expressed as the mean ± SEM (*n* = 4). After treatment, immunofluorescence staining was used to determine α-SMA expression (green) (**B**,**E**) and stress fiber formation (red) (**C**,**F**). Nuclei were stained with DAPI (blue). Scale bar, 10 μm. Data are expressed as the mean ± SEM (*n* = 3). * *p* < 0.05 vs. vehicle; ^#^ *p* < 0.05 vs. TGF-β1.

ET-1 is necessary to complete Ang II-mediated cardiac hypertrophy [28]. TGF-β and ET-1 cooperate in the pathophysiology of lung fibrosis [29]. Thus, Ang II-mediated ATR signaling is complex and involved in a cascade of molecular interactions of TGF-β1 and ET-1. As shown in Figure 3, blockade of ETRs using bosentan significantly inhibited TGF-β1-induced collagen I and α-SMA synthesis and stress fiber formation. In contrast, blockade of ATRs with valsartan did not attenuate profibrotic effects of TGF-β1 (Figure 3A–C). These results indicated that TGF-β1 is an important downstream effector of ATRs and exerts fibrotic effects through ETR signaling to drive the transformation of fibroblasts into myofibroblasts. Hence, ET-1 is a downstream effector of TGF-β signaling.

### 2.5. Smad and ERK Are Required for TGF-β1-Induced Myofibroblast Differentiation in Adult HCFs

The canonical Smad-dependent pathway and non-canonical MAPK-dependent pathway have been implicated in TGF-β signaling and have been shown to contribute to the development of fibrosis in various tissues [30]. Therefore, we investigated whether these targeted proteins of TGF-β1 signaling are involved in the regulation of myofibroblast differentiation in adult HCFs by using the specific inhibitors SIS3 (Smad3 inhibitor), FR180204 (ERK1/2 inhibitor), and SB203580 (p38 MAPK inhibitor). Pretreatment with either SIS3 or FR180204 significantly suppressed TGF-β1-induced overexpression of collagen I and α-SMA, including stress fiber formation (Figure 3D–F). Contrarily, pretreatment with SB203580 did not show inhibitory effects on TGF-β1-mediated myofibroblast differentiation. Interestingly, dual inhibition of Smad3 and ERK1/2 activities completely blocked these TGF-β1 effects (Figure 3D–F). Hence, TGF-β1 transduces fibrogenic signaling through canonical Smad and noncanonical ERK1/2 cascades in adult HCFs.

### 2.6. ET-1 Is a Downstream Effector of Ang II/TGF-β Axis That Stimulates Myofibroblast Differentiation via the ET_A_R Subtype in Adult HCFs

**Figure 4:**
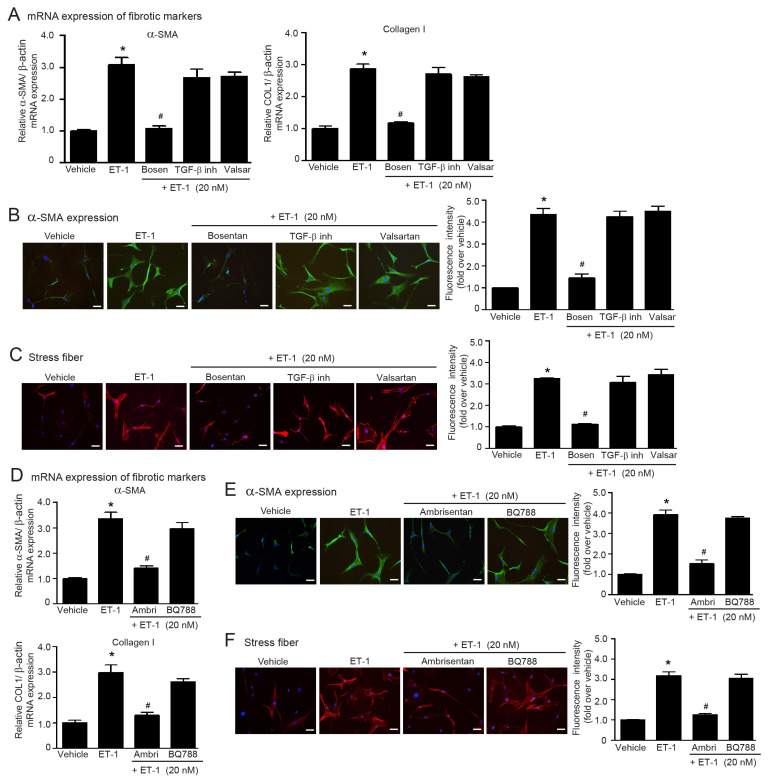
ET-1 acts as a downstream effector of Ang II and TGF-β1, which induces α-SMA and collagen I expression and stress fiber formation through the ET_A_Rs. (**A**–**C**) Serum-starved adult HCFs were incubated with LY2109761 (TGF-β inh; 5 µM), bosentan (1 µM), or valsartan (valsar; 1 µM) for 1 h before stimulation with 20 nM ET-1 for 6 h (**A**) or 24 h (**B**,**C**). (**D**–**F**) Serum-starved adult HCFs were pretreated with 1 µM ambrisentan (ambri; ET_A_R antagonist) or 1 µM BQ788 (ET_B_R antagonist) for 1 h before stimulation with 20 nM ET-1 for 6 h (**D**) or 24 h (**E**,**F**). (**A**,**D**) Relative mRNA levels of fibrotic markers, α-SMA, and collagen I were analyzed by qRT-PCR. Data are expressed as the mean ± SEM (*n* = 4). After treatment, immunofluorescence staining was used to determine α-SMA expression (green) (**B**,**E**) and stress fiber formation (red) (**C**,**F**). Nuclei were stained with DAPI (blue). Scale bar, 10 μm. Data are expressed as the mean ± SEM (*n* = 3). * *p* < 0.05 vs. vehicle; ^#^ *p* < 0.05 vs. ET-1.

TGF-β induced ET-1 synthesis in vascular endothelial cells [14,15] and blockade of ET_A_Rs abolished Ang II-increased ET-1 contents in aorta and femoral artery [19]. Thus, ET-1 is a downstream effector of Ang II and TGF-β1 in the vascular system. We further tested whether ET-1 acts as a downstream effector of the Ang II/TGF-β axis in myofibroblast differentiation in adult HCFs. We found that the antagonism of ETR with bosentan completely suppressed ET-1-stimulated α-SMA and collagen I synthesis and stress fiber formation (Figure 4A–C). Blockades of either TGF-β receptors with LY2109761 or ATRs with valsartan did not inhibit the profibrotic effects of ET-1 (Figure 4A–C), indicating that ET-1 serves as a downstream effector of ATR and TGF-β receptor signaling. 

In addition, ET-1 binds to ETRs, including ET_A_R and ET_B_R, and exerts its effects on tissue fibrosis [31]. Here, we also determined the subtype specificity of ETRs in ET-1-induced myofibroblast differentiation in adult HCFs. Pretreatment of fibroblasts with ambrisentan (ET_A_R antagonist), but not BQ788 (ET_B_R antagonist), was able to antagonize ET-1-induced α-SMA and collagen I synthesis and stress fiber formation (Figure 4D–F). Collectively, ET-1 acts as a downstream target of the Ang II/TGF-β1 cascade by binding to and activating the ET_A_R subtype to promote myofibroblast differentiation.

### 2.7. G_αq_-Dependent Activation of AT_1_Rs Is Necessary for the Upregulation of TGF-β1 and ET-1 in Adult HCFs

**Figure 5:**
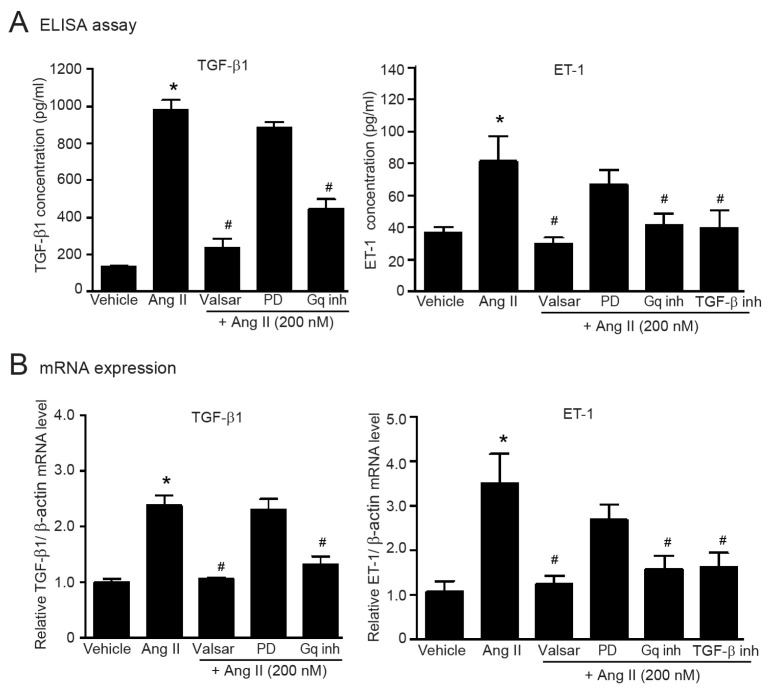
Ang II induces the secretion and synthesis of TGF-β1 and ET-1 via the AT_1_R/G_αq_ axis in adult HCFs. Serum-starved adult HCFs were incubated with valsartan (valsar; 1 µM), PD123319 (1 µM), FR900359 (G_αq_ inh; 1 µM), or LY2109761 (TGF-β inh; 5 µM) for 1 h before stimulation with 200 nM Ang II for 24 (**A**) or 6 h (**B**). (**A**) After Ang II treatment, the culture medium was collected. The amounts of TGF-β1 and ET-1 secreted into the culture medium were determined by ELISA. (**B**) Relative mRNA levels of TGF-β1 and ET-1 were analyzed by qRT-PCR. Data are expressed as the mean ± SEM (*n* = 4). * *p* < 0.05 vs. vehicle; ^#^ *p* < 0.05 vs. Ang II.

TGF-β1 and ET-1 are the important effectors of ATR signaling; however, the subtype specificity of ATR on upregulation of TGF-β1 and ET-1 is unknown. We found that AT_1_R antagonism with valsartan completely abolished Ang II-induced secretion and synthesis of TGF-β1 and ET-1, whereas PD123319 had no effect on Ang II-mediated upregulation of TGF-β1 and ET-1 (Figure 5A,B). In addition, blockade of G_αq_ protein signaling with FR900359 significantly inhibited Ang II-mediated secretion (Figure 5A) and synthesis (Figure 5B) of TGF-β1 and ET-1. Moreover, pretreatment with LY2109761 (TβRI/II inhibitor) significantly suppressed Ang II-induced upregulation of ET-1 (Figure 5), indicating that the AT_1_R/TGF-β cascade is essential for ET-1 upregulation. Therefore, the AT_1_R/G_αq_ axis is necessary for upregulation of TGF-β1 and ET-1 in adult HCFs.

### 2.8. TGF-β1-Induced Upregulation of ET-1 Is Dependent of Smad and ERK1/2 in Adult HCFs

**Figure 6:**
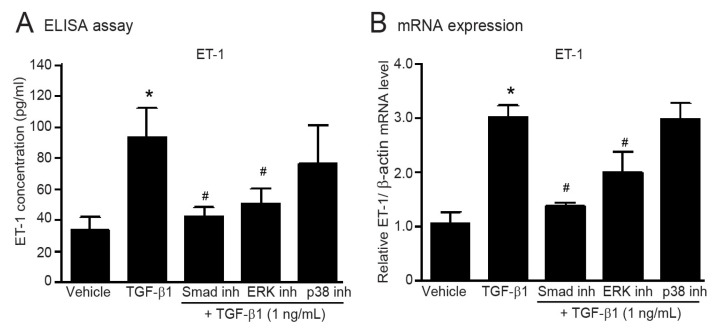
TGF-β1 induces the secretion and production of ET-1 via Smad- and ERK1/2-dependent pathways**.** Serum-starved adult HCFs were pretreated with 1 µM Smad inhibitor (Smad inh), 1 µM FR180204 (ERK inhibitor; ERK inh), or 1 µM SB203580 (p38 MAPK inhibitor; p38 inh) for 1 h before treatment with 1 ng/mL TGF-β1 for 24 (**A**) or 6 h (**B**). (**A**) After TGF-β1 treatment, the culture medium was collected. The amounts of ET-1 secreted into the culture medium were determined by ELISA. (**B**) Relative mRNA levels of ET-1 were analyzed by qRT-PCR. Data are expressed as the mean ± SEM (*n* = 4). * *p* < 0.05 vs. vehicle; ^#^ *p* < 0.05 vs. TGF-β1.

Since TGF-β-induced ET-1 mRNA expression is dependent of Smad proteins in vascular endothelial cells [16], we next examined the possible role of downstream mediators of TGF-β signaling on upregulation of ET-1 in adult HCFs. The secretion and synthesis of ET-1 potentially increased following TGF-β1 stimulation. In contrast, TGF-β1-mediated ET-1 upregulation was attenuated in the presence of either SIS3 (Smad inhibitor) or FR180204 (ERK1/2 inhibitor), while inhibition of p38 MAPK had no effect (Figure 6). These results demonstrated that TGF-β1 induces upregulation of ET-1 through the canonical Smad-dependent and non-canonical ERK1/2-dependent pathways in adult HCFs. 

### 2.9. The Reversibility of Ang II-Induced Myofibroblast Differentiation by Dual Inhibition of TGF-β Receptor and ETR

**Figure 7:**
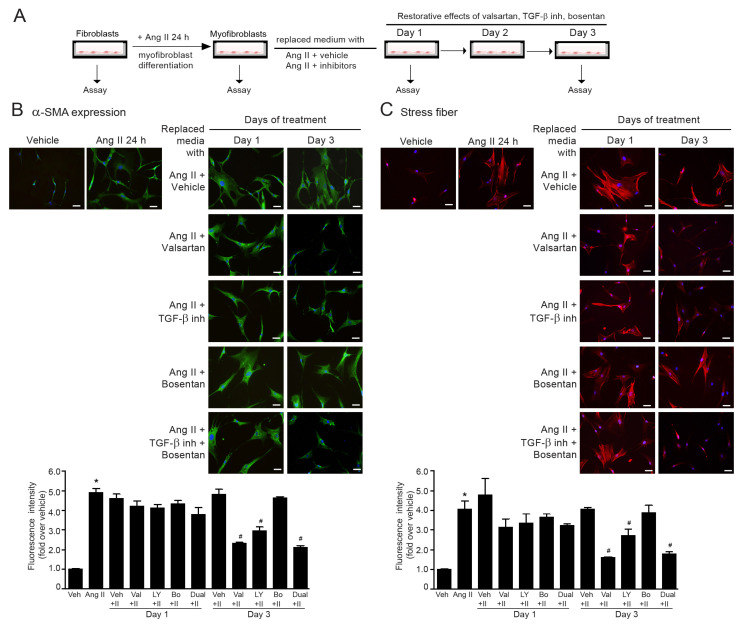
Restorative effects of ARB, TGF-β inhibitor, and ERA on Ang II-induced myofibroblast differentiation. (**A**) Experimental design and timeline of the treatment in cultured adult HCFs. (**B**,**C**) Fibroblasts were treated with 200 nM Ang II for 24 h to activate fibroblast-to-myofibroblast transformation. After 24 h, cells were replaced with media containing Ang II with different inhibitors, including valsartan, LY2109761, bosentan, and LY2109761 plus bosentan for up to 3 days. Analysis was performed on days 1 or 3 after cotreatment. At the end of treatment, immunofluorescence staining was used to determine α-SMA expression (green) (**B**) and stress fiber formation (red) (**C**). Nuclei were stained with DAPI (blue). Scale bar, 10 μm. Data are expressed as the mean ± SEM (*n* = 3). * *p* < 0.05 vs. vehicle; ^#^ *p* < 0.05 vs. Ang II.

Once cardiac fibrosis has already occurred, the reversal of myofibroblast differentiation is considered as a potential strategy to suppress the progression of cardiac fibrosis [32]. In this study, we investigated the effects of valsartan (ATR antagonist), LY2109761 (TβRI/II inhibitor), bosentan (ETR antagonist), and LY2109761 plus bosentan on the reversibility of myofibroblast differentiation induced by Ang II. Firstly, adult HCFs were incubated with Ang II (200 nM) for 24 h to induce fibroblast-to-myofibroblast transformation, which is characterized by α-SMA overexpression and actin stress fiber formation. After Ang II incubation and changing the culture medium, cells were co-treated with Ang II with vehicle, valsartan, LY2109761, bosentan, or LY2109761 plus bosentan, and cultured for up to 3 days. The assessments of α-SMA expression and stress fiber formation were performed on day 1 and day 3 of treatment (Figure 7A). In the absence of inhibitors (Ang II + vehicle group), Ang II induced an increase in α-SMA levels and stress fiber formation for up to 3 days of observation (Figure 7B,C). The 1-day incubation of all inhibitors was insufficient to reverse myofibroblast differentiation. However, on day 3 of observation, α-SMA-positive myofibroblasts and stress fiber formation were dramatically attenuated in the presence of either valsartan or LY2109761 (Figure 7B,C). Moreover, a combination of TβRI/II inhibitor with ERA reversed the myofibroblast phenotype, as demonstrated by a reduction in α-SMA expression and stress fiber formation (Figure 7B,C). The potency of dual inhibition of TGF-β receptor and ETR in the reversal of myofibroblasts was similar to that of valsartan. These data implied that dual blockade of TGF-β and ET-1 signaling can reverse Ang II-induced myofibroblast differentiation in adult HCFs.

## 3. Discussion

The transdifferentiation of fibroblasts into myofibroblasts is a key cellular mechanism in the heart that drives the fibrotic responses and ultimately contributes to myocardial fibrosis [32]. Myofibroblasts are smooth muscle-like active fibroblasts that overexpress α-SMA, contain large bundles of actin microfilaments organized into prominent stress fibers, and are the major source of ECM proteins in fibrotic hearts [5,33]. Regardless of the pathology of fibrosis, myofibroblast differentiation is a hallmark of the fibrotic response; therefore, agents capable of interrupting the transformation process could potentially prevent maladaptive tissue fibrosis and remodeling in response to profibrotic stimuli such as Ang II, TGF-β1, and ET-1. 

Stimulation of AT_1_Rs by Ang II promoted interstitial fibrosis, contributing to ventricle wall stiffness and cardiac dysfunction [6]. Contrarily, blockade of AT_1_Rs by ARBs resulted in the alleviation of cardiac fibrosis in rat models of pressure overload or myocardial infarction [34,35]. Consistent with these animal studies, our present study demonstrated that Ang II stimulates myofibroblast differentiation, as determined by collagen I and α-SMA overexpression, as well as stress fiber formation, in a dose-dependent manner via the AT_1_R subtype in adult HCFs. Antagonism of AT_1_Rs with valsartan prevented the fibrogenic effects of Ang II. AT_1_R stimulation leads to G protein coupling and dissociation of G*_α_*_q_ protein from G_βγ_ subunit [10]. Our previous study demonstrated that AT_1_R mediated upregulation of growth factors through G_αq_ protein, not G_αi_ or G_α12/13_ proteins [11]. In addition, G_βγ_ subunit of ATRs is also known to regulate the activities of targeted proteins. For instance, Ang II stimulated the Janus kinase 2/signal transducers and activators of transcription (JAK2/STAT) signaling via the G_βγ_ subunit in rat aortic smooth muscle cells [36]. In our present study, blockade of G*_α_*_q_ protein inhibited Ang II-induced myofibroblast differentiation, while blockade of G_βγ_ activity did not inhibit Ang II actions, emphasizing that the AT_1_R/G_α__q_ axis is necessary for the fibrogenic effects of Ang II in adult HCFs. 

TGF-β1 has been described as an essential mediator of RAS and plays an important role in the pathogenesis of remodeling heart. In TGF-β1 deficient mice, administration of Ang II did not promote myocardial fibrosis and hypertrophy [37]. Moreover, Ang II induced TGF-β1 expression in cardiac myocytes and fibroblasts, and upregulation of TGF-β1 was required for Ang II-induced hypertrophy [11,12]. In line with previous studies, blockade of TGF-β signaling with LY2109761 (TβRI/II inhibitor) significantly inhibited Ang II-induced myofibroblast differentiation. However, LY2109761 showed less inhibitory effects than valsartan. Thus, TGF-β1 is one of the downstream effectors of Ang II and acts as an important part of ATR signaling. TGF-β receptors are divided into two subtypes: TGF-β receptor type I (TβRI, or called ALK5) and type II (TβRII) [33]. TGF-β1 activates fibroblasts and other cells (e.g., epithelial cells, endothelial cells, and pericytes) to form myofibroblasts via two major signaling pathways, namely the canonical Smad-dependent and non-canonical MAPK-dependent pathways [33,38]. Ang II-induced collagen synthesis required TGF-β/Smad and MAPK signaling [39]. In addition, activation of ERK1/2 by Ang II is dependent on TGF-β [11]. The findings from our study demonstrated that inhibition of either Smad3 or ERK1/2 activities attenuate TGF-β1-stimulated myofibroblast differentiation. Therefore, TGF-β1 mediated myofibroblast transdifferentiation of adult HCFs through the Smad and ERK1/2 cascades.

ET-1 is one of the key players in the initiation and maintenance of tissue fibrosis and the effects of Ang II are partially mediated through the action of ET-1. ET-1 secreted from endothelial cells was required for Ang II-induced myocardial hypertrophy and fibrosis [28]. In addition, treatment with losartan prevented an increase in aorta ET-1 content induced by Ang II [20]. Our results showed that blockade of ET-1 signaling using bosentan alone tends to reduce Ang II-induced myofibroblast differentiation. Interestingly, dual antagonisms of ETR and TGF-β receptor signaling by co-treatment with bosentan and LY2109761 (TβR I/II inhibitor) potentially inhibited Ang II actions, exhibiting inhibitory potency similar to that of valsartan. Our data indicated that ET-1 appears to function synergistically with TGF-β1 to promote myofibroblast differentiation induced by Ang II. TGF-β and ET-1 have cooperative functions in the vascular system, particularly in endothelial cells. Despite these findings, the precise nature of the interaction between TGF-β and ET-1 in human cardiac fibroblasts remains incompletely understood. Our study demonstrated that ET-1, but not Ang II, is a downstream effector of TGF-β1 signaling. In addition, Ang II, TGF-β1, and ET-1 did not act independently from each other, but synchronously acted as part of a complex signaling network to induce myofibroblast differentiation. 

ET-1 is upregulated in hypertensive hearts, and the endothelin system played a critical role in the development of hypertrophy and fibrosis in a rat model of HF [40]. Although the detrimental effects of ET-1 are mostly mediated via ET_A_R, ET_B_R may play a part in the pathophysiological event of cardiac fibrosis since ET_B_Rs were detected to be upregulated in fibrotic-related diseases such as scleroderma-associated fibrotic lung disease [41] and pulmonary arterial hypertension [42]. Here, we demonstrated that ET-1-induced myofibroblast differentiation is suppressed by antagonism of ET_A_Rs, highlighting the role of ET_A_Rs in activation of human HCFs. 

There are several lines of evidence regarding the relationship between the RAS, TGF-β signaling, and endothelin system. For instance, Ang II mediated myocardial fibrosis by upregulating TGF-β1 synthesis in infarcted rat hearts [43]. Ang II induced mRNA expression of preproendothelin-1 [44] and increased the release of the mature ET-1 peptide in vascular smooth muscle cells [45]. Here, we demonstrated that Ang II induces synthesis and secretion of TGF-β1 and ET-1 through the AT_1_R/G*_α_*_q_ cascade. We also found that TGF-β1 is required for Ang II-induced ET-1 synthesis in adult HCFs. TGF-β1 potentially induced ET-1 upregulation, which required the cooperation of Smads and activator protein 1 (AP-1) in vascular endothelial cells [16]. On the other hand, another study reported that TGF-β-induced ET-1 production occurs through a Smad-independent ALK5/JNK/AP-1-dependent pathway in normal and fibrotic human lung fibroblasts [46]. Our findings provided evidence that TGF-β1 induces ET-1 synthesis and secretion in adult HCFs through both Smad-dependent and ERK1/2-dependent pathways, while Smad and ERK1/2 are likely involved in the upregulation of ET-1. Thus, further investigation is needed to fully understand the mechanisms and complexities of these pathways.

Prevention of myofibroblast differentiation by using specific inhibitors for signaling molecules has shown advantages in antifibrotic events. Previous studies demonstrated that myofibroblast differentiation could be reversed with anti-fibrotic agents such as prostaglandin E_2_ [47], metformin [48], and ERAs (ambrisentan and bosentan) [49]. As shown in this study, treatment with either valsartan or LY2109761 (TβRI/II inhibitor) promoted dedifferentiation of myofibroblasts after day 3 of treatment with reductions in *α*-SMA expression and stress fiber formation, emphasizing the restorative effects of ARB and TβRI/II inhibitor. However, treatment with bosentan alone could not show a significant reversal of this differentiated phenotype. Remarkably, dual inhibition of TGF-β receptor and ETR showed more efficacy in the reversibility of myofibroblast differentiation compared to TβRI/II inhibitor alone, highlighting current therapeutic strategies for limiting cardiac fibrosis, highlighting current trends in the approach to attenuating cardiac fibrosis. One limitation is that our results derived from in vitro experiments. Therefore, further investigations are necessary to explore the preventive and restorative effects of these specific inhibitors in animal models of fibrosis.

## 4. Materials and Methods

### 4.1. Reagents

Ang II, ET-1, PD123319, gallein (G_βγ_ inhibitor), and BQ788 were obtained from Tocris Bioscience (Ellisville, MO, USA). Recombinant human TGF-β1, valsartan, bosentan, ambrisentan, LY2109761, FR180204, and SB203580 were obtained from Sigma Aldrich (Saint Louis, MO, USA). SIS3 (Smad3 inhibitor) and FR900359 (G_αq_ inhibitor) were obtained from Cayman Chemical (Ann Arbor, MI, USA). Fibroblast growth medium and related cell culture reagents were obtained from Promocell (Heidelberg, Germany).

### 4.2. Fibroblast Cultures

Adult HCFs were obtained from Promocell (catalogue number C-12375). Cells were cultured in fibroblast growth medium and incubated at 37 °C in a humidified incubator containing 5% CO_2_. Fibroblasts from the second to fourth passages were used for all assays.

### 4.3. Immunofluorescence Staining for the Detection of α-SMA Expression

Adult HCFs (1 × 10^4^ cells/well) were seeded onto gelatin-coated cover slips in 12-well plates, and cells were allowed to attach overnight. After treatment, cells were fixed with 4% paraformaldehyde at 4 °C overnight. Cells were permeabilized by 0.1% Triton X-100 solution and blocked with 2% bovine serum albumin (BSA) for 1 h. Cells were then incubated with antibody against α-SMA (1:500, Sigma Aldrich), diluted in blocking buffer for 1 h and incubated with Alexa Fluor 488 secondary antibody (1:250, Invitrogen; Carlsbad, CA, USA) for 1 h. After washing with phosphate buffered saline (PBS), cells were mounted with antifade mounting medium containing 4′,6-diamidino-2-phenylindole (DAPI) (Invitrogen) [50]. The images were captured at 20× magnification by fluorescence microscope at wavelengths of 488/520 nm (Nikon Eclipse Ts2R; Tokyo, Japan). Three to five images with non-overlapping areas were taken per each treatment group. The mean fluorescence intensity was analyzed using Image J software (version 1.53k, NIH, Bethesda, MD, USA).

### 4.4. Immunofluorescence Staining for Detection of Actin Stress Fiber

Fluorescent-labeled phalloidin was used for detection of actin stress fiber in the cells. Fibroblasts (1 × 10^4^ cells/well) were seeded on gelatin-coated cover slips in 12-well plates overnight. After treatment, cells were fixed with 4% paraformaldehyde at 4 °C overnight. A 0.1% Triton X-100 solution was used to permeabilize the cells, and 2% BSA was further added to block the cells for 1 h. Cells were incubated with Alexa Fluor 594 phalloidin (1:100, Invitrogen) for 1 h and washed with PBS. An antifade mounting medium containing DAPI (Invitrogen) was used to mount the cells. The images were captured at 20× magnification by fluorescence microscope at wavelengths of 581/609 nm (Nikon Eclipse Ts2R). Three to five images with non-overlapping areas were taken per each treatment group. The mean fluorescence intensity was analyzed using ImageJ software (version 1.53k, NIH, Bethesda, MD, USA).

### 4.5. Quantitative Real-Time RT-PCR

Total RNA was extracted from fibroblasts using GeneJET RNA isolation kits (Thermo scientific, Waltham, MA, USA). The quantitation of mRNA expression was analyzed using real-time qPCR. Amplification reactions were performed using AriaMx Real Time PCR system and Brilliant III ultra-fast SYBR green qRT-PCR master mix (Agilent Technologies, Santa Clara, CA, USA). The isolation of total RNA and real-time qRT-PCR were performed as previously described [51]. All primer sequences were obtained from Macrogen (Seoul, Republic of Korea). The mRNA expression of COL1A1, α-SMA, TGF-β1, and ET-1 was determined using primers as described: COL1A1 (sense, 5′-CTGCTGGACGTCCTGGTG AA-3′; antisense, 5′-ACGCTGTCCAGCAATACCTTGAG-3′), α-SMA (sense, 5′-TGGCTATTCCTTCGTTACTACTGCT-3′; antisense, 5′-CATCAGGCAACTCGTAACTCTTCTC-3′), TGF-β1 (sense, 5′-CCCAGCATCTGCAAAGCTC-3′; antisense, 5′- GTCAATGTACAG CTGCCGCA-3′), and ET-1 (sense, 5′-GACATCATTTGGGTCAACACTC-3′; antisense, 5′-GGC ATCTATTTTCACGGTCTGT-3′). The ratio of the mRNA of interest was normalized with β-actin (sense, 5′-GTGGCCGAGGACTTTGATTG-3′; antisense, 5′-AGTGGGGTGGC TTTTAGGATG-3′). Relative gene expression was determined by the 2^−ΔΔCT^ method. 

### 4.6. Enzyme-Linked Immunosorbent Assays (ELISA) 

Adult HCFs (5 × 10^5^ per/well in 6-well plate) were treated with specific reagents in serum-free medium for 24 h. After treatment, the culture medium was collected and stored at −80 °C for further use. The levels of TGF-β1 and ET-1 in the culture medium were measured by ELISA (TGF-β1 ELISA kit, ab100647; ET-1 ELISA kit, ab133030 from Abcam). Briefly, 100 μL of each standard and each sample (culture medium) was added to the appropriate wells as previously described [52]. After the end of reaction, samples were immediately measured for absorbance at specific wavelengths using an EZ Read 400 microplate reader (Biochrom, Cambridge, UK). The amounts of TGF-β1 and ET-1 in the culture medium were calculated using the standard curve as described in the manufacturer’s instructions. 

### 4.7. Quantification and Statistical Analysis

Data are presented as the mean ± SEM of 4 independent experiments. The statistical differences were analyzed by one-way ANOVA with Tukey’s post hoc test for comparisons of three or more groups and Student’s t-test for two groups. A *p* value below 0.05 (*p* < 0.05) was considered statistically significant. Statistical analysis was performed with GraphPad Prism (version 6.0).

## 5. Conclusions

**Figure 8:**
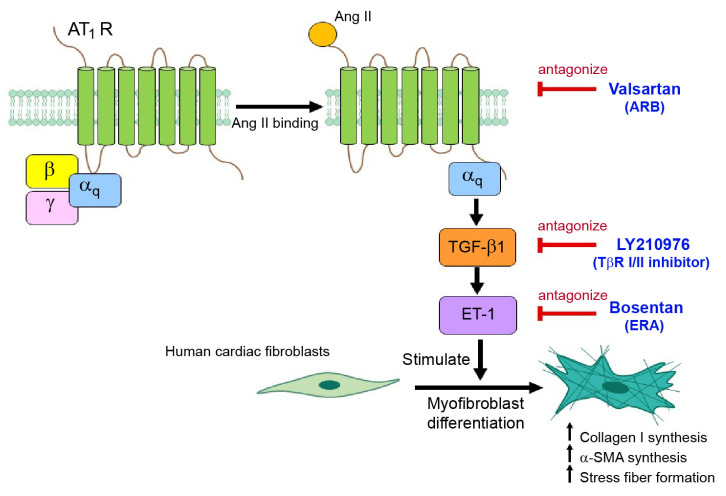
Schematic representing the TGF-β1, ET-1 and Ang II networking on myofibroblast differentiation. In adult HCFs, Ang II stimulation of AT_1_Rs lead to an upregulation of TGF-β1 and ET-1 and activation of their effectors through the AT_1_R/G**_α_**_q_ axis. The activated AT_1_Rs transduce the signal through TGF-β1 cascade, which in turn upregulates ET-1 through the Smad-dependent and ERK1/2-dependent pathways. The elevated ET-1 then binds to and activates ET_A_Rs, leading to increases in collagen I and α-SMA synthesis and stress fiber formation. Interestingly, antagonism of AT_1_Rs and dual blockade of TGF-β1 receptors and ETRs exhibit antifibrotic effects by prevention and restoration of myofibroblast differentiation induced by Ang II. α-SMA: α-smooth muscle actin; Ang II: angiotensin II; AT_1_R: angiotensin II receptor type 1; ARB: angiotensin II receptor blocker; ET-1: Endothelin-1; ERAs: endothelin receptor antagonists; TβR I/II: TGF-β receptor type I and II.

In summary, our data supported a concept whereby TGF-β1 and ET-1 are required for AT_1_R-stimulated myofibroblast differentiation in adult HCFs. Ang II induced myofibroblast differentiation via the AT_1_R/G_αq_ cascade (Figure 8). The activated AT_1_Rs transduced the signal through TGF-β1 cascade and consequently upregulated ET-1 via the Smad-dependent and ERK1/2-dependent pathways. Thus, TGF-β1 and ET-1 are downstream effectors of Ang II and act as part of the ATR signaling and network for myofibroblast differentiation. Interestingly, dual antagonism of TGF-β receptor and ETR could prevent and reverse the myofibroblast phenotype induced by Ang II. This study reported on a novel therapeutic strategy for the prevention and restoration of Ang II-mediated cardiac fibrosis.

## Data Availability

Raw data presented in this study are available on request from the corresponding author.

## Supplementary Materials

The following supporting information can be downloaded at: https://www.mdpi.com/article/10.3390/ijms24086972/s1.
