# Supplementary material for: Dual Blockade of TGF-β Receptor and Endothelin Receptor Synergistically Inhibits Angiotensin II-Induced Myofibroblast Differentiation: Role of AT1R/Gαq-Mediated TGF-β1 and ET-1 Signaling
Source: Int J Mol Sci. 2023 Apr 9;24(8):6972. doi: 10.3390/ijms24086972 (PMC10138810; doi:10.3390/ijms24086972)
Supplement: Supplementary file 1 [file ijms-24-06972-s001.zip › ijms-2294894-SI.pdf]

# Supplementary information

## Supplementary figure S1

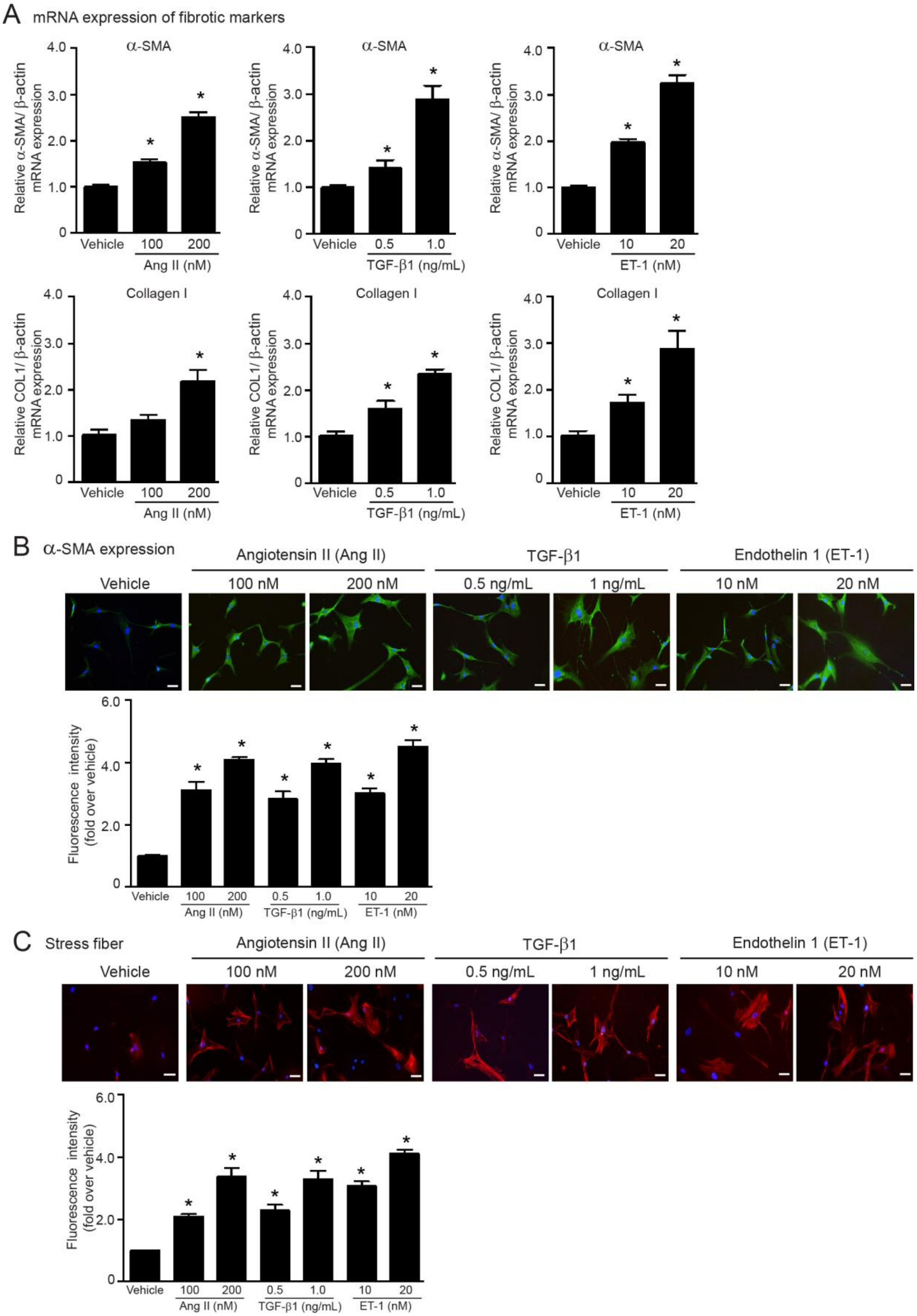

**Supplementary Figure S1. Treatment with Ang II, TGF-β1, or ET-1 induced myofibroblast differentiation in a dose-dependent manner**

Serum-starved fibroblasts were incubated with different doses of Ang II (100 and 200 nM), TGF-β1 (0.5 and 1 ng/mL), and ET-1 (10 and 20 nM) for 6 h (A) or 24 h (B-C). (A) Relative mRNA levels of fibrotic markers, α-SMA and collagen I were analyzed by real-time qRT-PCR. After the treatment, immunofluorescence staining was used to determine α-SMA expression (green) (B) and stress fiber formation (red) (C). Nuclei were stained with DAPI (blue). Scale bar represents 10 μm. *; $P < 0.05$ vs. vehicle. $N = 3$ and data were presented as the mean ± SEM.
